# Retraction Note: The LINC00623/NAT10 signaling axis promotes pancreatic cancer progression by remodeling ac4C modification of mRNA

**DOI:** 10.1186/s13045-023-01506-5

**Published:** 2023-11-01

**Authors:** Zengyu Feng, Kexian Li, Kai Qin, Juyong Liang, Minmin Shi, Yang Ma, Shiwei Zhao, Huaiyu Liang, Dongni Han, Baiyong Shen, Chenghong Peng, Hao Chen, Lingxi Jiang

**Affiliations:** 1 Department of General Surgery, Pancreatic Disease Center, Research Institute of Pancreatic Diseases, Ruijin Hospital, Shanghai Jiao Tong University School of Medicine, Shanghai, 200025 People’s Republic of China; 2 Shanghai Key Laboratory of Translational Research for Pancreatic Neoplasms, Shanghai, People’s Republic of China; 3 Institute of Translational Medicine, Shanghai Jiao Tong University, Shanghai, People’s Republic of China; 4 Department of General Surgery, The Second Affiliated Hospital, School of Medicine, Zhejiang University, Hangzhou, People’s Republic of China; 5 Otolaryngology and Head and Neck Center, Cancer Center, Department of Head and Neck Surgery, Zhejiang Provincial People‘S Hospital (Affiliated People‘s Hospital, Hangzhou Medical College), Hangzhou, Zhejiang People’s Republic of China; 6 Key Laboratory of Endocrine Gland Diseases of Zhejiang Province, Hangzhou, Zhejiang People’s Republic of China; 7 Department of Pathology, Ruijin Hospital, Shanghai Jiao Tong University School of Medicine, Shanghai, People’s Republic of China; 8 State Key Laboratory of Analytical Chemistry for Life Science, School of Chemistry and Chemical Engineering, Nanjing University, Nanjing, People’s Republic of China

**Retraction to:  Journal of Hematology & Oncology (2022) 15:112** **https://doi.org/10.1186/s13045-022-01338-9**

The Editor-in-Chief has retracted this article because of significant concerns regarding a number of Figures presented in this work, which question the integrity of the data. The Editor-in-Chief therefore no longer has confidence in the integrity of the data in this article.

Lingxi Jiang does not agree to this retraction. Zengyu Feng, Kexian Li, Kai Qin, Juyong Liang, Minmin Shi, Yang Ma, Shiwei Zhao, Huaiyu Liang, Dongni Han, Baiyong Shen, Chenghong Peng and Hao Chen have not responded to any correspondence from the editor about this retraction.

